# Development and Use of the Leadership Competencies for Healthcare Services Managers Assessment

**DOI:** 10.3389/fpubh.2019.00034

**Published:** 2019-02-28

**Authors:** Cynthia A. Hahn, Marisa Gil Lapetra

**Affiliations:** ^1^Department of Member Services, American College of Healthcare Executives, Chicago, IL, United States; ^2^International Hospital Federation, Bernex, Switzerland

**Keywords:** healthcare management, leadership, global health, competency directory, self-assessment

## Abstract

Healthcare management as a profession continues to evolve as the field of healthcare delivery becomes more complex. In some countries, formal degree programs in healthcare management and related professional associations have helped to establish the field as a distinct profession with a defined body of knowledge. In other countries, the professionalization of healthcare management has not developed, or is in the early stages of development. Many of these Low and Middle Income Countries (LMIC) have no formal training programs or professional associations to help set and define minimum standards and competencies for the profession. In many countries, national associations have been created by healthcare managers for sharing knowledge, information, and expertise. While major differences exist in the contexts where healthcare managers operate around the world, all have a common responsibility to enhance the leadership and managerial capacity and capability of their membership as well as promote the profession they represent. In spite of these efforts by national professional organizations and various ministries of health, healthcare management has not been universally recognized around the world as a profession. The *Leadership Competencies for Healthcare Services Managers* (Global Competency Directory) framework developed by the International Hospital Federation's global consortium for healthcare management serves as a catalyst and resource for defining the skills, knowledge, and abilities needed for the healthcare management profession. This article documents the purpose, development, validation, and use of the framework.

## Background

Healthcare management as a profession continues to evolve as the field of healthcare delivery becomes more complex. In some countries, formal degree programs in healthcare management and related professional associations have helped to establish the field as a distinct profession with a defined body of knowledge. In other countries, the professionalization of healthcare management has not developed, or is in the early stages of development. Many of these Low and Middle Income Countries (LMIC) have no formal training programs or professional associations to help set and define minimum standards and competencies for the profession.

In many countries, national associations have been created by healthcare managers for sharing knowledge, information, and expertise. In some countries, associations have a formal mandate to represent the profession, while in other countries they exist as *ad hoc* groups without regulatory or legal authority. While major differences exist in the contexts where healthcare managers operate around the world, all have a common responsibility to enhance the leadership and managerial capacity and capability of their membership as well as promote the profession they represent (1). In spite of these efforts by national professional organizations and various ministries of health, healthcare management has not been universally recognized around the world as a profession. The Global Competency Directory framework developed by the International Hospital Federation's Global Consortium for Healthcare Management, serves as a catalyst and resource for defining the skills, knowledge, and abilities needed for the healthcare management profession. This article documents the purpose, development, validation, and use of the framework.

### Global Consortium for Healthcare Management Professionalization

In 2013, the leaders of organizations^1^ representing government, the private sector, healthcare associations, and academic institutions met to respond to the challenge of raising the recognition of and promoting the professionalization of healthcare management globally by developing a Global Competency Directory. Completed in 2015, the Directory represents core competencies required of healthcare managers regardless of position, setting, or country (2).

The shared value of all participants is professionalizing leadership and management of health systems globally. To further promote these shared values and enhance the leadership and management practices in healthcare, the Global Consortium for Healthcare Management Professionalization was created and is recognized and supported by the International Hospital Federation (2).

### Creating a Charter to Guide the Mission

To guide the strategy, a Charter was developed for the professionalism of healthcare management and from there to develop an international agreement on core competencies for healthcare managers who lead healthcare organizations.

The Goals of the Charter included:

- To professionalize the Healthcare Management discipline
- To build global capacity in the leadership and management of health systems for healthy communities

Objectives:

- To develop an internationally agreed upon set of core competencies for healthcare managers to possess
- To use the core competencies framework as a fundamental tool to strengthen the training, employment and promotion of healthcare managers
- To encourage healthcare planners and human resource managers to develop long-term career pathways for healthcare managers and leaders in the health sector
- To promote the formalization and acceptance of healthcare management associations within countries and regions, thus allowing for peer control and development

## Developing the Global Competency Directory

The directory development process used available documentation, subject matter expert (SME) workshops, and a survey of international healthcare management experts to describe the requirements of healthcare managers among different countries including Low and Middle Income Countries (LMIC) and healthcare delivery settings. The work of the Global Consortium for Healthcare Management Professionalization began in 2012. An initial group of 12 subject matter experts from international organizations^2^ met for a 2-day workshop and completed a cross-walk of current competency management frameworks around the world. These included the United States' Healthcare Leadership Alliance Competency Model (3, 4), Canada's Canadian College of Health Leaders LEADS model (5), Australia's competency model developed by Health Workforce and supported by the Australasian College of Health Service Management (6), the United Kingdom's competency model developed by the NHS Leadership Academy (7), the Pan American Health Organization's (PAHO) Core Competencies for Public Health framework (8), and the emerging country's competency model developed by Management Sciences for Health for the United States Agency for International Development (9–11).

**Figure 1:**
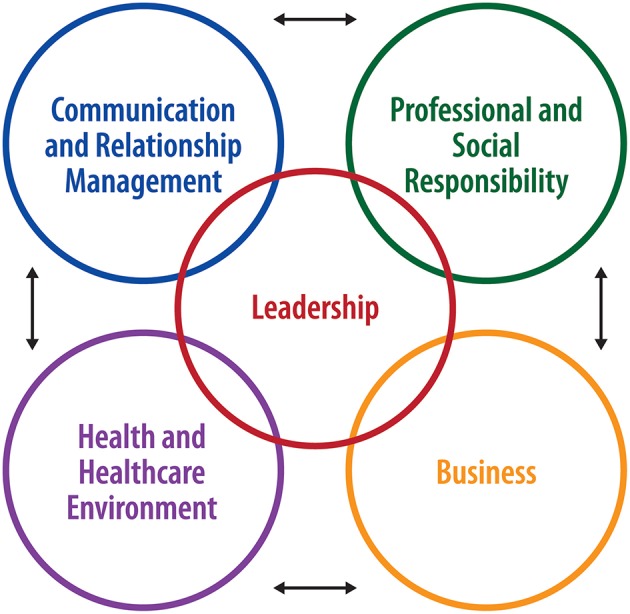
Global competency model domains.

After reviewing the various competency models, the SMEs agreed to develop a global competency framework patterned on the United States' Healthcare Leadership Alliance (HLA)^3^ competency directory. The HLA competencies were developed from job analysis surveys conducted to determine the relevant tasks performed by healthcare managers regardless of work setting or years of experience, and are clustered around five major domains (see Figure 1).

**Figure 2:**
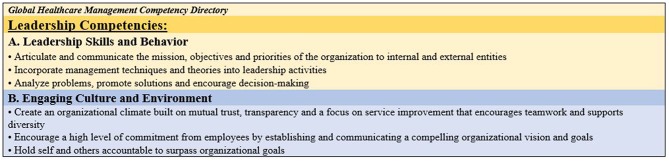
Sample competency statements from the leadership domain.

A sample of competency statements from the Leadership domain are presented in Figure 2 below.

In January 2013, the group of SMEs met to achieve agreement on the fundamental competencies needed for health managers, the target audience for the framework, the appropriate range of competencies, and the target audience for the directory. To develop the list of competencies in the model, the group first reviewed the competencies in the HLA model and selected those that are universal regardless or country or work setting. From this preliminary list,they drafted a list of knowledges, skills, and abilities (KSAs) to reflect competencies needed globally. In early 2013, the KSA lists were reviewed and revised by several SMEs via web conference calls. These lists were again reviewed and finalized by a group of SMEs during a face-to-face workshop. The second half of 2013 was spent reviewing and formatting the initial competency directory.

## Validating the Directory

After the group reached consensus on the competencies, the initial directory was comprised of over 300 competency statements, organized around five domains (Leadership, Communication and Relationship Management, Professional and Social Responsibility, Health and Healthcare Environment, and Business). It was sent in survey format in November and December 2013 to experts from a variety of healthcare management settings and locations throughout the world, including those in government, associations, academia, and healthcare management (see Table 1). The experts reviewed the directory, gave feedback, and ranked the competencies according to importance for the healthcare management settings in their countries.

**Table 1:** Demographics of survey respondents.

**Country/ Region**	***N***	**(%)**
Australia	3	3.1
Africa	3	3.1
Asia	3	3.1
Austria	2	2.0
Belgium	5	5.0
European Union	44	45
Hungary	2	2.0
Latin America	7	7.2
Norway	3	3.1
Singapore	2	2.0
Slovakia	1	1.0
Switzerland	4	4.1
United Kingdom	1	1.0
United States	16	16
**SETTING**		
Hospital	34	35.4
Hospitai/Healthcare Association	20	20.8
Ministry of Health/Government	3	3.1
Other	31	32
University	8	8.0
**TITLE**		
President/CEO	30	31
Director	25	26
Manager	9	9.3
Professor	4	4.1
Consultant	1	1.0
Other	27	28

In January 2014, the group expanded to 22 SMEs representing 15 different countries. The group reviewed survey responses and used the rankings to identify KSAs required of competent managers at any level in their career and regardless of delivery setting or country.

Using the feedback and rankings from the survey respondents, a small working group continued consolidating the number of competencies down to a core group of KSAs. The final version of the directory consists of 80 core healthcare management competencies grouped into the five major domains represented in Figure 1.

In January 2015, the full group of 22 SMEs met in-person for the third time to review the updated Competency Directory, move forward with a call to action, and develop a communications plan.

## Call to Action

A *Call to Action*, made at the June 2015 International Hospital Federation Meeting in Chicago, called for governments and the international health community to advocate for the worldwide utilization of the core competencies framework in the training, employment, and evaluation of healthcare managers and leaders (2).

The Consortium calls for the adoption of the Global Competency Directory as the initial basis for healthcare management development frameworks and programs, for use by academic institutions and relevant licensing, and accrediting bodies.

The Consortium recognizes that the competency framework must remain flexible and needs to allow adaptation for specific circumstances of each country. Accordingly, the competencies identified in the directory may be adapted to ensure their relevance in the local environment.

Recognizing the need for greater progress in the ongoing effort to build professional healthcare management capacity, the members of the Consortium agree that the following measures should be implemented according to national circumstances and needs (2):

- Adoption of the Global Competency Directory to inform and align healthcare management development programs at all levels of undergraduate, postgraduate, and ongoing education and professional development
- Customization and incorporation of each of the competency requirements into formal credentialing systems, which should be based on independent evaluation and evidence of demonstrated competencies
- Formal recognition at the national level of healthcare management as a profession
- Implementation of merit-based career advancement along with a career path for healthcare managers and leaders
- Recognition of healthcare managers' professional associations as key stakeholders for policy dialogue related to leadership and management and for the advancement of the profession

The need for urgency is based on the realization that advances in healthcare depend on the professional management of healthcare organizations and continuous improvement of competencies for healthcare managers.

## Implementation

### The Global Competency Directory

The International Hospital Federation, together with the Consortium, undertook and led considerable efforts to enhance the professionalization of healthcare management. Adopting this comprehensive list of core competencies is critical to enhance leadership and managerial capacities of healthcare managers. The Consortium formed the Healthcare Management Special Interest Group (HM SIG), which has since adopted action plans to effectively implement the acceptance and appropriation of the core competencies by professional associations worldwide. Promoting the systematic use of the Global Competency Directory is acknowledged as key to improving healthcare management. The Healthcare Management Special Interest Group SIG has been promoting the Directory among healthcare management professional associations along with online the self-assessment instrument.

### The Development of the Competency-Assessment Platform

The Healthcare Management and Leadership Competency Assessment Platform was created based on the hard copy Global Competency Directory. It is an online assessment instrument available free of charge to any healthcare professional in a management position. The platform is available in Chinese, English, Farsi, French, Spanish, and Portuguese. The Competency Assessment instrument was designed to promote the Global Competency Directory and thereby enhance leadership for managers, by measuring potential improvements. This online instrument acts as a guide for healthcare managers in planning, developing, and implementing concrete steps along their professional career, through continuous professional development. For instance, the tool may be used to identify an individual's strengths and/or weaknesses from the set of core competencies encouraging the individual to reflect on their competencies and create a future plan for professional development.

Participants' confidentiality, security, and protection of information are assured by Swiss regulations and laws. The tool consists of a two-step survey of 80 multiple-choice questions, one per competency.

Structurally, the survey is designed as a dual exercise. First, to rate the relevance of each of the competencies as assigned by the user, and second, to allow the user to self-rate his or her skills with respect to the same competencies. The tool was developed to ensure randomization of questions to avoid self-gaming as much as possible. To that end, while the content of each section was kept together, the order of questions was randomized. Keeping the exercise self-directed was the primary objective. Dividing the survey into two sub-surveys provides additional benefit to the user for added insight and reflection of an individual's competency level. In return, users receive a double set of results showing margin for improvement and personal gaps compared to average values collected from peers worldwide.

Two different five-point scales are used to collect user responses in each sub-survey. However, it was necessary to keep the same format while using a different nomenclature. Both are standard five-point scales. The relevance scale goes from *1* = *not relevant for my current position in my current organization* to *5* = *very relevant to my current position in my current organization*. The individual competency scale uses the Dreyfus model going from 1 = Novice; 2 = Advanced beginner; 3 = Competent; 4 = Proficient; and 5 = Expert (12). It was widely agreed that each of the 80 competencies was important to a healthcare manager's skill; therefore, the survey does not leave the “not applicable” option in either scale. The exercise was conceived as a comprehensive evaluation of an individual's skill level over the 80 competencies within the Global Competency Directory.

The competency assessment is a thorough exercise in self-introspection and as such the platform was designed to be as user-friendly as possible. For instance, it is easy to log in and out of the assessment tool as many times as wished. A memory-based algorithm is embedded in the tool, which saves a user's answers. Users are advised to take their time when answering the questions and to try not to remember responses provided in the first part. The goal of the exercise is for users to compare the relevance attributed to each competency and the level of self-reported proficiency, thereby determining the potential competency gap(s) for improvement. At the end of the two-step survey, the user receives a report summarizing the outcomes of the test, and identifying gaps between attributed relevancy and score for each question. Through the two scores used to qualify each competency, the final report offers an at-a-glance visualization for every competency's gap. For example, if the end-user considered “*Human Resource Management” as* “**very relevant”** for his or her current position, but admitted being only “**competent**” the report would display a need for improvement in this competency.

On a second level, by consolidating results from other end-users, and taking into account the central limit theorem (CLT)^4^ to account for confidentiality, the platform delivers a real-time and objective comparative report. End-users can thus look at peers (by function, by position, by professional experience, or by location) and compare responses for each competency. This may encourage self-reflection, such as “*What can I learn from this result?”* The platform provides an anonymous comparison between individuals, based on the individual profile information that was required to open a user account.

Future versions of the platform will recommend online resources for end-users to utilize as they plan their career progression. Being open resourced, the IHF Healthcare Management SIG encourages other professional associations worldwide to participate in the initiative by contributing their country-specific material to the directory. The platform builds on an online repertoire of multicultural/multi-language educational resources open to anyone willing to undergo the survey. In this way the platform would not only supply resources to support continuous professional development but, warrant and assure an exchange of valuable resources regularly updated. Resources are curated and validated by the IHF Healthcare Management SIG prior to their publication.

Finally, by agreeing to contribute on an organizational level, the platform becomes a unique reference tool assisting healthcare management associations, as well as governments and organizations in identifying gaps in management and leadership skills of healthcare management professionals. In the future, the IHF Healthcare Management SIG endeavors to analyze and correlate leadership and management skills to hospital performance outcomes.

## How it is Being Used

### Individual Use

Individually, end-users are only requested to open an account and fill out a set of questions regarding their professional status, such as positions, education, and experience. Personal information, which is never disclosed, pertains to age, country of origin, and gender. These basic demographic data are necessary to qualify groups of peers and perform adequate comparisons. The survey was taken anonymously by over six hundred healthcare managers in the first few months following its activation, regardless of their professional link to the IHF or any national healthcare organization.

The IHF Healthcare Management SIG has prompted an intensive use of the tool among regional groups. Neighboring countries sharing common concerns realized that they could use the tool to jointly explore trends. As was mentioned earlier, the Consortium recognizes that the competency framework must remain flexible and needs to be adaptable for the specific circumstances of each country to ensure its relevance in the local environment. Discussions with these regional groups of countries confirmed their interest in examining consolidated outcomes to evaluate regional patterns of competencies. Below are a few questions to be assessed by regional groups:

1. Do the same management competencies apply?
2. Are different competencies needed in these countries?
3. Do competencies improve performance in non-similar settings?
4. What is the most effective way to teach and train competent managers in these countries?

And some other questions were brought up around the future development of competencies:

- Are formal healthcare management training (degree programs) needed to develop management competencies?
- Are degree programs effective for developing competent managers?
- At what levels should intervention/training occur to best develop management competencies?

Currently, these questions have propelled an intense debate among the Mediterranean countries including Spain, Italy, Portugal, and Greece. Future plans for other regional groupings include:

- Latin-American countries: Colombia, Argentina, and Brazil
- East-African countries: Kenya, Uganda, Ethiopia, Tanzania
- East-European countries: Poland, Hungary, Moldova, Romania, and Ukraine

Future developments will focus on Low and Middle Income Countries (LMIC) to determine what competencies are needed and how this differs from high-income countries through research and data analysis.

### Organizational and National Use

On an organizational and national level, the self-assessment of personal leadership, and management competencies in healthcare delivery organizations is viewed as a way to adapt and modify healthcare programs and to encourage continuous professional development across organizations. Regional and national groups add momentum to open up dialogue with policy- and decision-makers.

The Directory is being used by several countries as a basis for healthcare management educational content for training programs and graduate and postgraduate degree programs. For example, the Catalan College of Healthcare Management (Societat Catalana de Gestió Sanitària) is working to incorporate the Global Healthcare Management Competencies as a basis for healthcare management training for future healthcare managers (14). The Loma Linda University School of Public Health is also using the competencies to develop curriculum and training sessions for a leadership certificate designed for leaders in 14 Mexican and Central American hospitals. Additionally, the Australasian College of Health Service Management (ACHSM) used the Directory in revising their own competency framework and in accrediting university healthcare management programs. The Royal College of Surgeons in Ireland (RCSI) Institute for Leadership is using the Directory to develop educational offerings (15).

Future plans include specific analytic work and further academic development, including a dictionary of healthcare executive competencies. Currently, the IHF Healthcare Management SIG has created an open dialogue with healthcare program education and accreditation bodies to further the work already in place.

## Future Plans

The International Hospital Federation has offered related national healthcare associations the opportunity to work together by sharing and discussing results of survey data. The sharing of information and results will provide useful information both on an individual and organizational basis for different professional profiles, their expectations, and how these vary across positions and/or geographical regions. Ultimately, professional profiles could be mapped against a matrix of competencies and compared to outcome indicators on an operational perspective. On top of it all, the tool intends to facilitate a roadmap by establishing the evolution of skills, encouraging improvement in areas highlighted as weak, and providing adequate input or resources. The self-assessment tool implies self-awareness, provides comparison, evaluation, and suggests recommendations, both at an individual and at an organizational level.

The Global Competency Directory and the online Leadership Competency Assessment Platform are offered to the public and remain flexible and adaptable to the relevance of each country. The IHF is not prescriptive in how the competencies are used, but hope that the competencies will be the catalyst for the development of healthcare management frameworks in countries where they do not exist, and to promote the professionalization of healthcare management globally. In countries, institutions, educational settings, or accrediting agencies where competency frameworks already exist, the Global Competency Directory and Leadership Competency Assessment Platform are meant to stimulate discussion and fill gaps, similar to how the Australasian College of Health Services Management used the framework in revising its own model. Countries and organizations are free to adapt the framework to their individual needs without approval of the IHF, however IHF staff are available as a resource to answer questions and offer suggestions on the process. As more countries and organizations use the framework, the IHF will collect a repository of examples to serve as additional resources to those who wish to use it. The IHF will also collect articles and case studies on how the framework is being used in different settings, and will post these to the IHF website as they become available.

By providing a community-wide knowledge hub, the Healthcare Management and Leadership Competency Assessment Platform contributes to building a high-level ranking of factual needs in the professionalization of healthcare managers regardless of an individual's geographic location or responsibilities.

## Author's Note

The title of the Global Healthcare Management Competency Directory document is *Leadership Competencies for Healthcare Services Managers*, for simplicity it is referred to as the Global Competency Directory in this article.

The Global Competency Directory was developed into an interactive online self-assessment platform called the Healthcare Management and Leadership Competency Platform. It is referred to as the online Competency Assessment Platform in this article.

## Author Contributions

CH is a subject matter expert in the development of healthcare management competencies. She was a major contributor to the development and production of the Global Competencies Directory, and authored sections Background, Developing the Global Competency Directory, Validating the Directory, and Call to Action of the article. MG is an expert in health economic policy evaluation and assessment. She worked on the development of the self-assessment instrument and with guiding countries on its use. She authored sections on Implementation, How it is Being Used, and Future Plans of the article.

### Conflict of Interest Statement

The authors are employed by the American College of Healthcare Executives CH and the International Hospital Federation MG.

## Footnotes

^1^International Hospital Federation, Pan American Health Organization, American College of Healthcare Executives, Australasian College of Health Service Management, Canadian College of Health Leaders, Taiwan College of Healthcare Managers, Health Management Institute of Ireland, European Association of Hospital Managers, Jamaican Association of Health Services, Management Sciences for Health, International Health Services Group, THET Partnership for Global Health, Sociedad Chilena de Administradores en Atención Medica y Hospitalaria,Federación Andina y Amazónica de Hospitales, Federacion Latinoamericana de Hospitales, University of the West Indies, Federacao Brasileirade Administradores Hospitalares, Hong Kong College of Healthcare Executives (2).

^2^This group is a subset of experts from the organizations listed in footnote 1.

^3^In addition to the American College of Healthcare Executives, other members of the Healthcare Leadership Alliance (HLA) are American Association for Physician Leadership, American Organizations of Nurse Executives, Healthcare Financial Management Association, Healthcare Information and Management Systems Society and the Medical Group Management Association.

^4^In statistics, the theorem stating that if a series of data sets is drawn from any probability distribution, the distribution of the means of those data sets will follow a normal distribution (13).
